# Innovative Integrated Motivational Interviewing for Dual Management in Tuberculosis Patients with Diabetes (MID-DOT) in Malaysia

**DOI:** 10.3390/healthcare11131929

**Published:** 2023-07-04

**Authors:** Zahiruddin Wan Mohd, Siti Rohana Ahmad, Nor Azwany Yaacob, Noorsuzana Mohd Shariff, Mat Zuki Jaeb, Zalmizy Hussin

**Affiliations:** 1 School of Medical Sciences, Universiti Sains Malaysia, Kota Bharu 16150, Malaysia; drzahir@usm.my; 2 State Health Department of Kedah, Alor Setar 05400, Malaysia; drsitirohana@gmail.com; 3 Advanced Medical and Dental Institute, Universiti Sains Malaysia, Gelugor 11800, Malaysia; suzanashariff@usm.my; 4 Hospital Raja Perempuan Zainab II, Kota Bharu 15586, Malaysia; 5 School of Applied Psychology, Social Work and Policy, Universiti Utara Malaysia, Sintok 06010, Malaysia; zalmizy@uum.edu.my

**Keywords:** tuberculosis, diabetes mellitus, dual management, motivational interview

## Abstract

(1) Background: Achieving successful tuberculosis (TB) treatment outcomes among diabetic patients is a real challenge as TB complicates control of diabetes. This study aimed to evaluate the effectiveness of an integrated dual management educational module, MID-DOTS, which uses the mmotivational interviewing (MI) technique implemented within directly observed treatment as part of a short course (DOTS) program in TB patients with diabetes (TB/DM). A randomized controlled trial was conducted in the northeastern state of Malaysia. (2) Methods: One hundred and twenty-four TB patients with diabetes received educational intervention using a MID-DOT module that used the MI technique, which was repetitively applied by TB nurses throughout a 6-month DOTS program while another 122 patients were given standard health education. Study outcomes include the proportion of patients with successful TB treatment, and changes in HbA1c and diabetic self-care scores at 6 months. (3) Results: The successful TB treatment outcome was 88% in the intervention group versus 72% in the control group (RR = 1.24; 95%CI 1.16, 1.58). A significant reduction of HbA1c (mean difference 0.82%; 95%CI 0.66, 0.98) and significantly higher diabetes self-care score (mean difference 8.49; 95%CI 7.38, 9.59) were also shown in the intervention group. (4) Conclusions: A dual TB/DM educational strategy which integrates the MI technique applied repetitively within the DOTS program is effective in increasing successful TB treatment as well as improving diabetic outcomes.

## 1. Introduction

An estimated 450 million people have diabetes worldwide and this number is anticipated to reach 630 million people by 2040 [1]. In Malaysia, the prevalence of known diabetes was 9.4% (95%CI: 8.66, 10.20) and the prevalence of raised blood glucose among those not known to have diabetes was 8.9% (95%CI: 7.96, 9.93) [2] in 2019. With the advent of diabetes, there has been a dramatic resurgence of tuberculosis (TB), as both diseases are linked at the rate of 10–30% [3]. Recent evidence has shown that the relative odds of developing TB are higher in diabetics than in non-diabetics, with odds ratios (OR) ranging from 2.44 to 8.33 [4,5,6,7]. A meta-analysis showed that the risk of death during TB treatment (RR 1.89, 95%CI; 1.52–2.36) and relapse following treatment (RR 3.89, 95%CI; 2.43–6.23) were both higher in TB patients with diabetes (TB/DM). The combined outcomes of TB treatment failure and death were also higher (RR 1.69, 95%CI; 1.36–2.12) [8].

Dual management is an option to achieve favorable outcomes for both diseases. Good glycemic control that may improve TB treatment outcomes requires pharmacological and non-pharmacological efforts from patients and healthcare providers [9]. The therapeutic regimens of TB and diabetes medications recommended by the WHO are highly effective, but non-adherence to treatment calls for attention [10] despite the success of the directly observed therapy (DOT) strategy for TB.

Non-adherence to treatment strategies has been identified as one of the barriers to achieving successful TB treatment outcomes and good glycemic control [11,12,13]. Type 2 diabetes, as a lifestyle disease, is influenced by multiple risk factors, thus adherence to lifestyle modification as part of self-care helps achieve good glycemic control instead of a prolonged treatment regimen [9]. Health personnel play a crucial role in improving patients’ knowledge, self-care, and internal motivation to improve the patients’ adherence to treatment. Their competency in delivering advice, style of counseling, and their frequent meetings with patients might influence patients’ desire to make sustained behavioral changes [14].

An appropriate style of counseling, repetitive education session, and person-delivered health education have been recognized as major factors that may influence patients’ motivation to make a behavior change [15]. The current practice of the traditional counseling approach often is forced, and instructs the patients to change without notice of the conflicts within the patient, which may lead to poor motivation to be responsible for their health [16]. Ideally, this message should be delivered repetitively, especially during six months of TB treatment [17].

Malaysia has followed the WHO recommendation of daily visits for a 2-month intensive phase followed by weekly visits for a 4-month period. The weekly visit is to supply a week of medication and for monitoring [18]. In the current Malaysian context, although TB clinic staff are engaging with patients during DOT, they are not trained as diabetes educators. The task of giving diabetes self-care education in TB/DM management is usually given to nutritionists and diabetes educators who only see patients for 3–6 months’ duration in diabetes clinics. In addition, there are limited educational materials or guidelines specifically addressing diabetes education for patients with TB. Although these materials are available, their complexity may lead patients to struggle in understanding the self-care needed to manage their chronic conditions, written medication, and translating the knowledge received into daily practice [17,19].

Motivational interviewing (MI) is defined as “a directive, client-centered counseling style for eliciting behavior change by helping clients to explore and resolve ambivalence” [16]. It is an evidence-based, collaborative, guided approach to health behavior change that emphasizes patients’ autonomy while building self-efficacy [20,21]. This approach has been used in smoking cessation and promoting medication adherence among TB patients [22]. Thus, the MI approach may shed new light in seeking to promote medication adherence and improve the dual management of TB/DM patients who struggle to fight infectious disease and chronic disease simultaneously.

This study addressed the research gap in alternative strategies for the non-pharmacological management of TB patients with diabetes. This study aimed to evaluate the effectiveness of an integrated strategy using motivational interviewing in the dual management of TB with diabetes. The TB primary outcome was treatment outcomes measured as the percentage of successful TB treatment. The secondary outcome was HbA1c and diabetes self-care measurement scores. The findings will delineate a dual management strategy that combines a TB educational module blended with lifestyle modification to facilitate TB health staff in simultaneously influencing the self-care of a patient facing two diseases.

## 2. Materials and Methods

### 2.1. Design

This study was a cluster randomized controlled trial of an innovative integrated strategy in the dual management of TB patients with diabetes. Motivational interviewing in diabetes and tuberculosis education within the directly observed therapy (MID-DOT) module facilitated TB nurses in influencing the self-care of patients facing two diseases. As soon as TB treatment was initiated, educational intervention using MI technique was given repetitively to TB patients with diabetes concurrently with their DOT program. The duration of intervention was the 6 months of their anti-TB regimen course.

### 2.2. Sample Size, Randomization, and Blinding

The sample size was determined by a two-proportion, independent samples formula to compare the proportion of successful TB treatment outcomes between the intervention and control groups. The sample size for comparing mean HbA1C and mean diabetes self-care score was estimated using PS Software 3.1 [23]. Estimation was carried out to achieve 80% power, a 5% significance level with consideration of the possibility of a 10% dropout rate.

Simple randomization was performed at the district level with a 1:1 allocation ratio generated using the random sequence number generator. The unit of randomization was the district, but the intervention program was specifically targeted at the patient’s level to avoid contamination of the intervention effect. A sampling of patients at the selected centers was not feasible due to the limited number of eligible patients, thus all eligible TB/DM patients from the centers were invited to participate. Since the trial was conducted in a routine healthcare setting, the allocation to intervention and control groups was not concealed from the top administrative person, TB nurses, patients, and assessor.

### 2.3. Recruitment of TB Treatment Center

TB treatment centers were defined as health centers that provide primary management of TB patients and offered directly observed therapy (DOT) programs with well-equipped laboratory and chest X-ray facilities and trained staff. These centers also managed TB patients with diabetes, including diagnosis, initiating, and reviewing the treatment of diabetes. Fifteen out of 25 TB treatment centers in intervention districts and 12 out of 20 in the control district group fulfilled the study criteria as TB treatment centers.

### 2.4. Study Participants

All TB patients with diabetes mellitus registered at and attending the TB treatment center in Kelantan from 1 January 2017 were screened for recruitment. Invitation to participate was offered to all adult patients above 18 years old. TB/DM patients were patients with bacteriologically confirmed pulmonary TB based on positive sputum smear for acid-fast bacilli [18] with old and new cases of type 2 diabetes mellitus (as diagnosed according to the type 2 diabetes diagnostic criteria [24] and received DOT at the TB treatment center. Poorly controlled TB/DM was based on a baseline HbA1c of more than 6.5%. TB patients with diabetes who were on regular hemodialysis, extrapulmonary TB, and drug-resistant TB cases who received different treatment regimens, as well as those with cancer and on chemotherapy, were excluded.

### 2.5. Study Duration

The module development commenced in September 2016 while patient recruitment started in January 2017. As soon as TB treatment was initiated, the intervention was given to individual patients concurrently with their DOT program. The duration of intervention was throughout their 6 months of the TB regimens course. Therefore, data collection was completed in November 2017 as the last patients were recruited in April 2017.

### 2.6. Intervention Module

The MID DOT intervention program was a structured education module to be delivered using the MI technique by the TB nurses who were involved with TB/DM patients in the DOT program. These included educational materials that specifically addressed TB and diabetes to enhance patient—TB DOT staff communication in the form of flip charts and posters. The educational materials were developed based on literature reviews and existing educational materials. Flip charts and posters were designed to achieve maximum comprehension by patients on an actionable plan not only to achieve TB treatment success but also diabetic control. Materials were validated by experts involved in TB and DM management and patient education. The TB nurses were trained prior to the intervention by research team members to use the MI to create a guiding, repetitive, and frequent diabetes education during patients’ visits to the TB clinic for DOT. Posters were pasted at the clinic for patients to view while waiting for the DOT activity.

### 2.7. Outcome Measurement Tools

#### 2.7.1. Malay Elderly Diabetes Self-Care Questionnaire (MEDSCaQ)

The questionnaire was developed and validated by Ishak et al. (2017) to assess diabetes self-care activities among TB patients with diabetes. It has five sections, on dietary control, physical exercise, self-monitoring blood glucose, medication adherence, and situational related adherence behavior, with factor loadings of 0.727–0.895, 0.925–0.930, 0.876, 0.634–0.809, 0.821–0.825, respectively. The MEDSCaQ has an overall Cronbach’s alpha of 0.721 and a range of 0.686 to 0.869 for the five subscales. The MEDSCaQ items were scored using 4-point scales, ranging from 0 to 3 where ‘0’ means ‘never’, ‘1’ means ‘seldom’, ‘2’ means ‘frequent’, and ‘3’ means ‘always’. The sum scoring was the summation of all 16 items giving the possible minimal score 0 and the maximum score 48. The higher the score, the better the self-care [25].

#### 2.7.2. Malaysian Version Michigan Diabetes Knowledge Test (MDKT)

The 14 items translated into Malay, and validated Michigan Diabetes Knowledge Test (MDKT) by Al-Qazaz et al. (2010), were used to measure diabetes knowledge among diabetes patients. The results had a good internal consistency (Cronbach’s alpha = 0.702) with a test—retest reliability value of 0.894 (*p* < 0.001). Subjects who scored less than 7 were in the low knowledge group, those who scored 7 to 10 were in the acceptable knowledge group, and those who scored 11 or more belonged to the good knowledge group [26].

#### 2.7.3. Questionnaire on Knowledge, Attitude, and Practice of Primary Care Providers on the Usage of Clinical Practice Guideline (CPG) on Type 2 Diabetes Mellitus

The 26-item self-administered questionnaire measured the knowledge, attitude, and practice of primary healthcare workers on the usage of diabetic CPG. There were two knowledge domains; screening and management of diabetes consisted of 10 items. The attitude domain consisted of 7 items and the practice domain consisted of 9 items. The questionnaire had a good internal consistency 78 (Cronbach’s alpha of all domains was above 0.7) [24].

### 2.8. Intervention Training and Standardization

The MID-DOT training program consisted of a one-day training workshop and a one-day field training session. Participants were 15 TB nurses who ran the TB clinic at the selected intervention TB treatment centers. Activities included lectures on the clinical practice guidelines of diabetes mellitus and tuberculosis, an introduction to MI, and diabetes self-care activities. It was followed by an interactive discussion on good dietary habits, physical activity, smoking cessation, self-blood glucose monitoring, and adherence to medication. A demonstration on flip chart usage and role play on MI skills were also included. Each session took about 30 min. One-day field training focused on hands-on application to a real patient at a TB clinic under facilitator supervision. The Motivational Interviewing Treatment Integrity coding system (MITI 4) form was used to evaluate the fidelity of MI. The advantage of the MITI 4 is its friendliness to raters who are not experts in MI, thus it does not require clinical proficiency in MI. The form consists of both global rating counts that consist of two technical components (Cultivating Change talk, and Softening Sustain talk) and two relational components (Partnership and Empathy), which are scored on a Likert-type scale from 1 (low competency) to 5 (high competency). An average score of 4 and above demonstrated good competency [27].

One-day field training was conducted at TB treatment centers. It was intended to help the TB nurses build their confidence in delivering TB and diabetes education to patients. Demonstrations by researchers regarding flip chart usage and counseling to the TB nurses were conducted. The TB nurses were then required to choose their own patients and practice the MI empathic counseling skills in front of the researcher. All 15 trained nurses passed the training with a minimum average score of 4 in each component.

### 2.9. MID-DOT Intervention

MID-DOT intervention consisted of interpersonal face-to-face health education on diabetes care which was integrated with DOT visits at the TB clinic for TB/DM patients during their weekly visits. The education was delivered by the trained TB DOT nurses using the standardized MID-DOT module. The education session took about 30 min and was conducted repetitively in one session per week with a total of 24 sessions during the entire length of 6 months of TB treatment. Education and advice were focused on lifestyle modifications including good eating habits, being physically active, and stopping smoking. Patients were guided to perform self-blood glucose monitoring and in good practice on daily medication intake. The MI was used to elicit the patient’s internal motivation for sustainable behavior changes. Cessation support for referral to diabetic educators, quit-smoking clinics, and pharmacists was also provided.

### 2.10. Quality Assurance

Besides training the TB DOT nurses of the intervention group to standardize the use of the MID-DOT module, a baseline assessment of knowledge, attitude, and practice using the MDKT questionnaire was conducted for nurses of both groups to ensure comparability at the baseline level. Fifteen TB nurses from 15 TB treatment centers (intervention) attended the workshop. TB nurses from 12 treatment control centers were at their treatment centers and distributed the questionnaire. The MDKT was repeated on the 6th month after the intervention period for both groups for reassessment and comparison.

### 2.11. Data Collection

Patients’ sociodemographic and clinical characteristics, comorbidity, presence of BCG scar, Mantoux test, laboratory test, HIV status, chest X-ray findings, and practitioners’ documentation were obtained from Tuberculosis Information System (TBIS), a standard national reporting system for TB cases. DOT activities and TB treatment outcomes were also extracted from the system. TB treatment outcomes were categorized into cured, completed, treatment failed, treatment interrupted, and died based on the national guidelines for TB and WHO recommendations [18]. Diabetes management information on body mass index, diabetes history and medication, and glucose monitoring during 6 months of TB treatment were extracted from the standard diabetes treatment book. All information was collected as the baseline for participants from both groups. Baseline HbA1c and MEDSCaQ questionnaire responses were collected for both groups.

Patients from the intervention group received education using the MID-DOT module while the control group received usual care, where TB education was given by TB DOT staff during patients’ visits to the TB clinic and diabetes education was delivered by the diabetic educator or general practitioners during routine appointments at a diabetes clinic, about 6 to 12 months apart. No shared responsibilities by TB DOT staff in delivering frequent diabetes education to patients and no standardized education module or materials were used for the control group.

**Figure 1:**
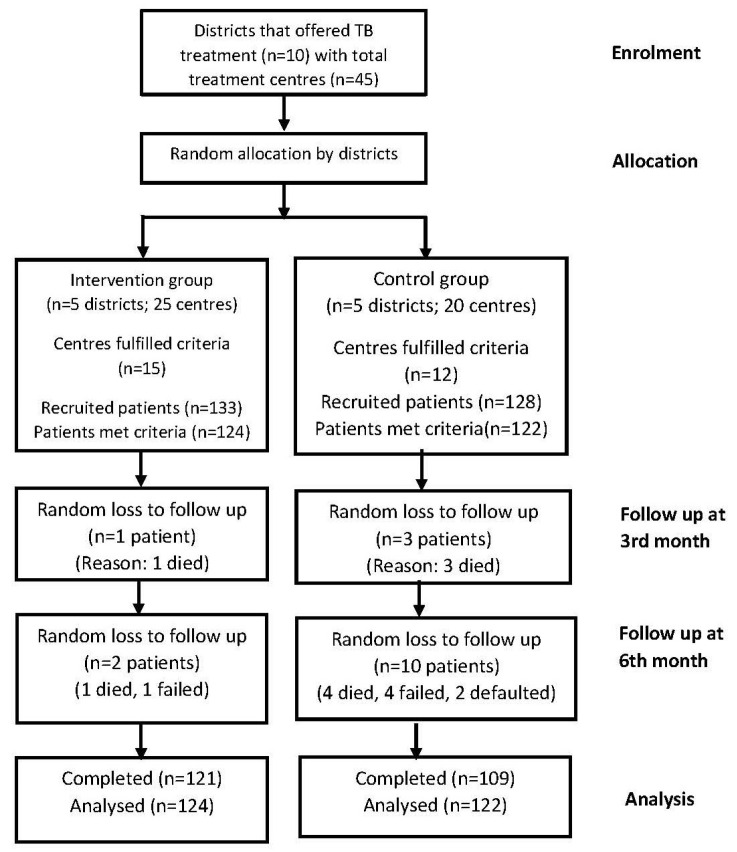
Flow chart of the study subject recruitment and data collection.

Participants were followed for up to 6 months of TB treatment. During the 3rd and 6th months of TB treatment, the HbA1c measurement and diabetes self-care activities MEDSCaQ were repeated. The TB treatments outcomes were evaluated during the 6th month of TB treatment (Figure 1).

### 2.12. Data and Statistical Analysis

Data entry and analysis were done using IBM SPSS version 22 software. Sociodemographic, clinical, and diabetes characteristics of all patients were tabulated. Univariable analysis was used to compare the baseline characteristics of patients between the intervention and control groups. The primary outcomes (percentage of successful TB treatment) were compared between two groups using a chi-square test with a significance target *p*-value < 0.005. The treatment effect between intervention and control groups was presented as relative risk (RR). Multiple logistic regression with adjusted OR was used to determine the factors associated with successful TB treatment outcomes among the TB/DM patients. The effect of the intervention on glycemic control and diabetes self-care activities based on the MEDSCaQ was determined by comparing the changes in the outcome measurement baseline at the 3rd month and 6th month of intervention in the control and intervention groups using repeated-measures ANOVA. Mauchly test and normality of numerical variables assumptions for the parametric tests were checked.

Ethical clearance approval for this study was obtained from the Malaysia Ministry of Health (MOH), Medical Research and Ethics Committee (MREC), and registered in the National Medical Research Register (NMRR) (NMRR-16-1996-32903). The study was also approved by the Human Ethics Committee, Universiti Sains Malaysia (USM/JEPeM/16100404). Written informed consent was obtained from each participant before data collection.

## 3. Results

### 3.1. Baseline Characteristics

Both intervention and control groups showed no significant difference in demographic characteristics. Subjects were predominantly males (77%), Malay (96%), and married (85%). The age ranged from 18 to 87 years old with more than half of patients aged 55 years and above with more than 5 years duration of illness. Most of them had received diabetes counseling before but the majority had it more than a year ago. Baseline knowledge and means of HbA1c and diabetes self-care activities (DSCA) scores did not show statistically significant differences (Table 1).

### 3.2. Effect of Intervention on TB Treatment Outcomes

Table 2 describes the comparison of TB treatment outcomes among intervention and control groups of study participants after the 6th month of TB treatment. The success of treatment for 109 patients in the intervention group was significantly higher (87.9%) compared with that of the 88 patients (72.13%) in the control group (*p* = 0.002). Relative risk (RR) for treatment success was 1.81 (95%CI: 1.16, 2.81).

Two significant independent factors contributed to successful TB treatment outcomes among TB patients with diabetes. The MID DOT intervention had nearly three times higher odds to have a successful TB treatment outcome compared to the standard separated patient education (adj OR 2.76, 95%CI:1.38, 5.49; *p* = 0.004). Additionally, the absence of DM complication also contributed to higher odds of successful TB treatment (adj OR 1.98, 95%CI: 1.01, 3.91; *p* = 0.047) (Table 3).

### 3.3. Effect of Intervention on HbA1c Measurement

**Figure 2:**
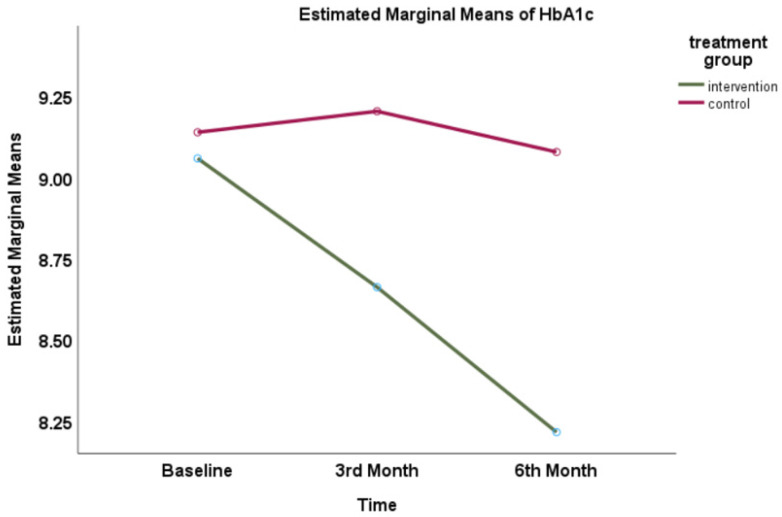
Profile plot of the mean HbA1c for each group for baseline, 3rd month, and 6th month of intervention.

Both groups had a reduction in their HbA1c level after the 6th month. However, a significantly higher reduction of HbA1c was observed in the intervention group (mean difference 0.45%, 95%CI: 0.34, 0.57). Repeated measures ANOVA showed a significant mean difference at baseline, 3rd, and 6th month within the group over time (time effect) as well as between the intervention and control groups (intervention effect). The baseline measurement was similar, and the measurement was significantly lower in the intervention group than in the control group. There was a time-group interaction effect. The partial eta square for the intervention effect was 0.19 and time effect was 0.15, indicating a large effect size (Figure 2).

### 3.4. Effect on Diabetes Self-Care Score Measurement

**Figure 3:**
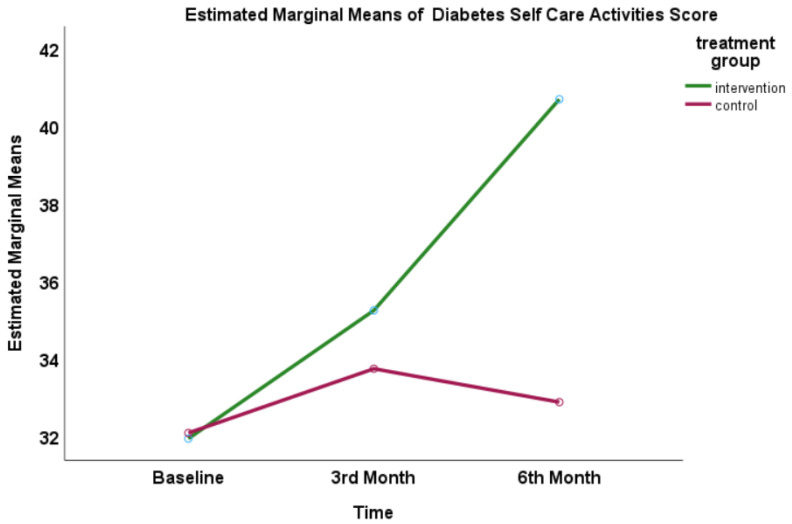
Profile plot of the estimated mean (estimated marginal mean) of mean DSCA score for each group for baseline, 3rd month, and 6th month of intervention.

There were significant mean differences between baseline, 3rd, and 6th month within the group over time as well as between the intervention and control groups. Both groups had an improvement in self-care scores after 6 months, but the improvement was higher in the intervention group (mean difference 3.01, 95%CI: 2.51, 3.59; *p* < 0.01). There was an interaction between groups and time (time-group interaction effect). The partial eta square for the intervention effect was 0.33 and time effect was 0.52, indicating a large effect size (Figure 3).

## 4. Discussion

The WHO has set the target rate for TB treatment success (percentage of patients who had been cured and completed a six-month TB course) at 85% as the national indicator for TB control programs [28]. The results obtained from this study proved that the MID-DOT intervention was effective for TB patients with diabetes, even after the adjustment for other confounders. This study used the MI technique with a structured educational module delivered during the patient’s TB DOT visit. ATB nurse educator met patients on a weekly basis for TB treatment and monitoring, integrating DM advice by educators familiar to the patient, plus the MI technique helped to improve not only TB treatment outcomes but also DM outcomes. MI techniques motivated patients to take action suitable for individual patient situations. This favorable outcome was consistent with the finding of a meta-analysis of 19 randomized controlled trial studies that determined the effectiveness of various strategies in improving TB treatment adherence and thus treatment outcomes among the TB population via an intervention program that focused on patients’ education combined with psycho-emotional counseling (RR 1.37; 95%CI 1.08–1.73) and socioeconomic support (RR 1.08; 95%CI 1.03–1.13). [29]. In addition, this finding was substantiated by a recent meta-analysis of nine randomized controlled trials on the effects of social protection, including the education intervention strategies on TB treatment outcomes in low- and middle-income countries [30]. It was revealed that the intervention was associated with TB treatment success (RR 1.09; 95%CI; 1.03–1.14) among patients with active TB. The findings from the two meta-analyses established a basis that educational intervention might improve TB treatment outcomes in lower-middle-income countries with a high burden of TB disease.

Previous studies have attempted to educate TB patients on treatment adherence and determined the effectiveness of their strategies in improving TB treatment outcomes. Thiam et al. had a similar design and method to this study, whereby a cluster-randomized trial was conducted at 16 government district health centers in Segal, France [31]. The study reported a high TB treatment success rate of 88% (RR 1.18; 95%CI 1.03–1.34) by improving the patients’ adherence using strategies such as reinforced counseling and repetitive DOT-based education delivered by the health personnel to the patients. Meanwhile, Bachmann et al. and Janmeja et al. similarly demonstrated that regular advice and education from health personnel could lead to more successful TB treatment outcomes among TB patients regardless of the mode of delivery [32,33].

In this study, education was effective even when almost half of the patients had a lower socioeconomic and educational status. The success of the intervention in this study could be attributed to the education approach. The MID-DOT intervention was targeted at the individual level, and MI-based TB/diabetes education was delivered face-to-face by the TB nurses. Although the individual education approach was less time-efficient than group education, it was more effective in enhancing the patients’ knowledge and practices, as group education often had poor attendance [34].

DOT was used as a platform for repetitive education in this study whereby patients were educated weekly for a total of 6 months. This repetitive education was the key to success of the intervention in this study. A meta-analysis revealed that contact with patients for more than 10 h could effectively improve patients’ knowledge and treatment adherence [35]. Another systematic review showed that when delivered repetitively, self-care education always resulted in good clinical outcomes regardless of the mode of delivery [36]. The treatment success of this study could be explained by the stronger patient—staff relationship resulting from their daily encounter with the same staff during DOT. Due to the established rapport, the TB patients were more likely to be in the receptive stage and tended to follow the instruction given by the TB nurses.

This study adopted a patient-centered approach to MI, which was delivered by the TB nurses in improving glycemic control, diabetes self-care practices, and TB treatment outcomes among TB patients with diabetes. As adherence declined due to a lack of motivation, MI could stimulate the patient’s internal motivation to adhere to the intervention strategies by exploring their ambivalence and supporting them to make better decisions [15]. The previous study, which assessed the feasibility of implementing brief MI in the context of TB treatment, found that the approach was feasible and practical to be used even for short patient visits during DOT [37]. Other studies reported that education delivered by chronic care staff, or a case management nurse equipped with education skills, decreased the HbA1c level and improved the patients’ knowledge and self-efficacy of patients, especially in diabetic patients with co-morbidities [21,38,39]. However, there are also studies that reported the failure of self-management education to improve patients’ HbA1c among diabetes patients when MI was delivered by diabetes educators or medical officers [40,41]. This may indicate that the frequent engagement of TB nurses with TB/DM patients through a DOT program may enhance the educational effectiveness in changing health behaviors.

In the present study, there was a statistically significant overall mean reduction of HbA1c levels in the intervention group at follow-up compared to the control group. The reduction also observed in the control group was expected, as in the usual practice, the physician will give more aggressive management when a patient has shown poor glycemic control during the TB treatment. The current study showed that a brief MI with the TB nurses was effective in enhancing the diabetes self-care practice and improving glycemic control among TB patients with diabetes. As shown by a similar study using cohort design by Pyan in Hawaii [41], this present study’s findings showed that repetitive DOT-based diabetes education could significantly improve patients’ glycemic control during the six-month TB treatment regardless of the mode of delivery.

Furthermore, the results of this present study were comparable with those of studies targeting the non-TB diabetes population. The findings of this study agreed with a systematic review of 120 randomized controlled trial studies, which reported a greater overall mean reduction in HbA1c (0.74) for patients randomized to diabetes self-care education as compared to that of the patients randomized to usual care (0.17) [37]. In addition, the magnitude of reduction in HbA1c among the participants exposed to diabetes self-care education exceeded that of those who received usual care by more than 0.5% for all modes of delivery. The review also reported an absolute improvement in HbA1c of 0.57, which was clinically significant and within the range of improvement observed with the addition of medications to a primary glycemic control treatment regime.

The results of this study were also consistent with the findings of a meta-analysis of 47 randomized controlled trials that reported 0.36% improvement in the glycemic control indexed by HbA1c in patients who received diabetes self-care education [35]. Despite the modest estimated reduction, evidence suggested that such a difference was significant enough to reduce the risk of developing diabetes complications. For instance, the United Kingdom Prospective Diabetes Study (UKPDS) revealed that a 0.9% decrease in HbA1c was associated with a 25% reduction in microvascular complications, a 10% decrease in diabetes-related mortality, and a 6% reduction in all-cause mortality [42].

An improvement in the diabetes self-care score was observed in the 6th month of intervention in both patient groups but the improvement was higher in the intervention group. This finding was similarly reported by Gabbay et al. [43], whereby the face-to-face structured education program improved the self-care practices among diabetic patients. Nevertheless, two other previous studies reported opposite findings, whereby the MI intervention had no impact on the patient-reported diabetes self-care behavior, with no statistical difference between the patient groups [44,45]. The comparison of this research work with the previous studies was limited because different questionnaires were used. Gabbay et al. [43] and Williams et al. [44] used the summary diabetes self-care activities (SDSCA) questionnaire, while Tan et al. [46] used the revised summary diabetes self-care activities (RSDCA) questionnaire. Both questionnaires analyzed the diabetes self-care elements in a separate domain while the present study used MEDSCAQ, which combined all domains into one score.

The improvement of self-reported diabetes self-care score in the control group of this study was as predicted. There were some plausible explanations: firstly, the brief advice received by the patients as part of the standard health education during their routine appointment at the diabetic clinic may have promoted good practice among them; secondly, answering questions regarding the diabetes self-care practices at baseline, 3rd month, and 6th month intervention period may have led to a reflection about the importance of glycemic control and subsequently altered their health behavior; lastly, being ill with TB may have increased their desire for better health, therefore motivating them to practice a better lifestyle.

This study had several strengths. Firstly, to the best of the researcher’s knowledge, no other study has evaluated a non-pharmacological intervention among TB patients with diabetes in Malaysia. This study would be the first randomized controlled trial that evaluated the effectiveness of the MI-based education approach implemented in the DOT program in improving glycemic control and TB treatment outcomes among TB patients with diabetes. Furthermore, this study also provided insight into the integrated TB and diabetes services in managing TB patients with diabetes. Secondly, the MID-DOT intervention was pragmatic in its design, utilizing resources that were readily available within the health centers. Lastly, this trial also successfully filled in an information gap and provided evidence on non-pharmacological management to enhance the glycemic control of TB patients with diabetes during the 6 months of TB treatment.

Nevertheless, the findings should be interpreted with caution due to several limitations. Firstly, the fidelity of the intervention could not be monitored by videotaping the actual MI sessions with the real patients as it would be considered invasive to the patients within the context of standard clinical care. Therefore, the possibility of a TB nurse, despite being intensively trained, failing to deliver the MI adequately could not be ruled out. However, such a possibility would be small considering the outcomes are similar or even better than what would have been expected with MI. Secondly, an intervention program focusing on different elements of diabetes self-care practice could not be organized. The original intention of this research was to organize the dietary intervention (food diary), the physical activity program such as exercise class, and the use of a pedometer was precluded by the logistic and financial conditions. As a result, a brief verbal self-efficacy approach was adopted instead of a structured dietary and physical intervention. Thirdly, the education intervention was about 30 min for each session to address all four self-care practices. Therefore, it was uncertain whether the participants in the intervention group understood their daily calorie intake and practiced self-care activities. Furthermore, the outcomes of good diabetes self-care practices were evaluated using a questionnaire only. However, this study was still acceptable as it was a pragmatic trial that focused on ways to implement an intervention in the daily clinical work of the general practice. Fourthly, the proportion of measurements differed between the intervention group and the usual care group although the baseline characteristics were well-balanced in all variables measured.

## 5. Conclusions

MID-DOT intervention delivered by TB nurses during DOT sessions as an adjunct to the standard health education was shown to be effective in increasing successful TB treatment outcomes among TB patients with diabetes as well as achieving good glycemic control and diabetes self-care practices. Engaging TB nurses in managing TB patients with diabetes for repetitive diabetes self-care education has the potential to reduce demands on diabetic educators, dieticians, and health services by providing additional support and potentially reducing the unsuccessful TB treatment outcomes rate.

## Figures and Tables

**Table 1:** Baseline comparison between intervention and control groups (*n* = 246).

Clinical Characteristics	Frequency (%)			
	Overall (*n* = 246)	Intervention (*n* = 124)	Control (*n* = 122)	*p*-Value ^b^
BMI (kg/m^2^) ^a^	22.4 (2.8)	22.3(2.1)	22.5 (3.4)	0.621 ^c^
Sputum for AFB				
Negative	48 (19.5)	28 (22.6)	20 (16.4)	0.146
Positive 1+	139 (56.5)	73 (58.9)	66 (54.1)	
Positive 2+	34 (13.8)	15 (12.1)	8 (15.6)	
Positive 3+	25 (10.2)	8 (6.5)	5 (13.9)	
HIV status				
Negative	195 (79.3)	95 (76.6)	100 (82.0)	0.535 ^d^
Positive	4 (3.2)	4 (3.3)	8 (3.3)	
Not done	43 (17.5)	24 (20.2)	18.0 (14.8)	
Chest X ray findings				
Mild	173 (70.3)	85 (68.5)	88 (72.1)	0.539
Severe	73 (29.7)	39 (31.5)	34 (27.9)	
Diabetes diagnosis				
Known case	214 (87.0)	112 (90.3)	102 (83.6)	0.117
Newly diagnosed	32 (13.0)	12 (9.7)	20 (16.4)	
Duration of diabetes				
Less than 5 years	67 (27.2)	36 (29.0)	31 (25.4)	0.0.523
5 years and more	179 (72.8)	88 (71.0)	91 (74.6)	
Diabetes complication				
No	156 (63.4)	82 (66.1)	74 (60.7)	0.373
Yes	90 (36.6)	42 (33.9)	48 (39.3)	
Last diabetes counseling				
Less than 1 year	81 (32.9)	41 (33.1)	40 (32.8)	0.963
1 year and more	165 (67.1)	83 (66.9)	82 (67.2)	
HbA1c level (%) ^a^	9.09(0.63)	9.05 (0.52)	9.14 (0.72)	0.316 ^c^
DM Knowledge score ^a^	6.71 (1.16)	6.75 (1.17)	6.67 (1.15)	0.599 ^c^
DSCA score ^a^	32.0 (2.86)	31.93 (3.02)	32.07 (2.69)	0.689 ^c^

^a^ Mean (SD), ^b^ chi-square test, ^c^ independent *t*-test, ^d^ Fisher exact test.

**Table 2:** TB treatment outcomes in intervention and control groups after 6 months (*n* = 246).

Treatment Outcomes	Intervention (*n* = 124)	Control (*n* = 122)	X^2^ Stat (df)	*p*-Value *
Success	109(87.90)	88(72.13)	8.252 (1)	0.002
Unsuccessful	15(12.10)	34(27.87)		

* chi-square test.

**Table 3:** Factors associated with successful TB treatment outcome among TB/DM patients.

Factors	Crude OR ^a^ (95%C1)	Adjusted OR ᵇ (95%CI)	Wald Statistic(df) ᵇ	*p*-Value ᵇ
Intervention group	2.81 (1.44,5.48)	2.76 (1.38,5.49)	8.37 (1)	0.004
No DM complication	2.12 (1.12,3.99)	1.98 (1.01,3.91)	3.95 (1)	0.047

ᵃ Simple logistic regression; ᵇ multiple logistic regression adjusted for occupation, education level, sputum grading severity, CXR severity, and duration of last counseling given. Hosmer and Lemeshow test, (*p* = 0.180); classification table (overall correctly classified percentage = 80.1%). Multicollinearity and interaction terms were not found. The area under ROC curve was (94%).

## Data Availability

The research data are available upon request.
